# Muscle changes with high-intensity aerobic training in an animal model of renal disease^1^

**DOI:** 10.1590/s0102-865020190050000003

**Published:** 2019-06-03

**Authors:** Eliane Barbosa Togoe, Iandara Schettert Silva, Juliana Loprete Cury, Flavia Alessandra Guarnier

**Affiliations:** I Fellow PhD degree, Postgraduate Program in Health and Development in Midwest Region, Universidade Federal do Mato Grosso do Sul (UFMS), Campo Grande-MS, Brazil. Conception and design of the study; acquisition, analysis, and interpretation of data; technical procedures; manuscript preparation and writing; critical revision.; II PhD, Associate Professor, School of Medicine, Postgraduate Program in Health and Development in Midwest Region, UFMS, Campo Grande-MS, Brazil. Conception and design of the study, acquisition of data, technical procedures, critical revision.; III PhD, Associate Professor, Centro Universitário da Grande Dourados (UNIGRAN), Dourados-MS, Brazil. Conception and design of the study, analysis and interpretation of data, manuscript preparation and writing, critical revision.; IV PhD, Associate Professor, Department of Pathological Sciences, Universidade Estadual de Londrina (UEL), Brazil. Technical procedures, histopathological examinations, analysis and interpretation of data.

**Keywords:** Renal Insufficiency, Chronic, Muscle Atrophy, Exercise, Rats

## Abstract

**Purpose:**

To analyze the muscle changes with high-intensity aerobic training (HIAT) in an animal model of renal disease (RD).

**Methods:**

Twenty one adult Wistar rats were divided into 3 groups: healthy sedentary (HS), RD sedentary (RDS), RD aerobic training (RDAT). RDS and RDAT were subjected to unilateral renal ischemia-reperfusion (10 min) and 21days after that, RDAT was subjected to 6 weeks HIAT (swimming). Serum creatinine (Cr) and muscle morphometry (cross-sectional area = CSA) of gastrocnemius were analyzed.

**Results:**

Cr was higher (p = 0.0053) in RDS (0.82 ± 0.04) than in the others (RDAT 0.55 ± 0.04; HS 0.55 ± 0.04). Morphometric analysis (class interval of CSA in μm^2^/absolute frequency of muscle fibers in each class) indicated that 50th percentile occurred in: HS 7th class (3000.00-3499.00/515), RDS, 8th class (3500.00-3999.00/484), RDAT 5th class (2000.00-2499.00/856). CSA of largest fibers in RDS, RDAT, HS was 9953.00 μm^2^, 9969.00 μm^2^,11228.00 μm^2^, respectively. High frequency of fibers with lower CSA occurred in 4th, 5th, 6th and 7th class in RDA, absence of fibers into 22nd, 23rd classes (RDS and RDAT).

**Conclusion:**

HIAT in an animal model of RD resulted in increased the number of muscle fibers with smaller CSA.

## Introduction

Chronic kidney disease (CKD) is a remarkable public health concern associated with an increased risk of mortality and comorbidities. It is estimated that 10%-15% of the general population is affected by CKD worldwide^1^.

Ischemia-reperfusion (IR) injury is the leading cause of CKD. In the kidneys, IR injury leads to acute and chronic complications that result in deficits in renal function and increased serum creatinine (Cr) levels due to a decrease in the glomerular filtration rate (GFR)^2^.

The GFR is used to classify renal disease (RD) in five stages (1-5) according to the degree of impairment of the kidneys. However, this classification does not provide parameters for assessing the functional effects on the other systems affected in the early stages of CKD development. The reduction in renal function leads to retention of uremic solutes, which causes inflammation, oxidative stress, and insulin resistance, thereby promoting the loss of muscle mass. Muscle atrophy is associated with cardiovascular disease, metabolic changes, and reduced physical function and balance, strongly impacting the quality of life and disease progression^3^.

Therapeutic strategies that help reduce the loss of muscle mass in CKD have been proposed, including aerobic training (AT). AT reduces the symptoms of uremia, inflammation, and oxidative stress markers, and improves skeletal muscle function, exercise capacity, and the quality of life of individuals. However, the effect of aerobic exercises depends on the load, type, and level of training^4^.

Analyzing the impact of AT on skeletal muscle is not feasible in clinical practice because it increases the complexity of the routine of renal patients. Therefore, animal models of disease are necessary to evaluate variables which cannot be adequately assessed in humans. This study aimed to analyze the muscle changes with HIAT in an animal model of RD.

## Methods

This study was approved by the Animal Research Ethics Committee, Universidade Federal do Mato Grosso (UFMS) under Protocol No. 735/2015.

Twenty-one albino Wistar rats (*Rattus norvegicus*) aged 53 days and weighing 200-250g were obtained from the UFMS animal facility. The animals were reared in cages lined with wood shavings, with four to five animals per cage, under controlled temperature (approximately 22ºC) and a light-dark cycle of 12/12h, with free access to water and commercial feed. The animals were randomly divided into three groups: a healthy sedentary (HS) group, rats with RD and sedentary (RDS), and rats with RD and subjected to AT (RDAT). The animals in the RDS and RDAT groups underwent surgery as per an IR procedure for induction of RD. The HS group was not subjected to surgery and received daily care.

The surgical protocol was performed under aseptic conditions according to the methodology proposed by Bazzano *et al.* ^5^ The animals were anesthetized with intraperitoneal 10% ketamine (50-100 mg/kg) and xylazine (1-5 mg/kg). Next, the animals were subjected to left flank laparotomy to locate the left kidney, which was externalized to remove the perirenal fat and isolate the pedicle. The renal artery and vein were clamped with non-traumatic forceps for 10 min and, during this period, ischemia was visually confirmed by a color change in the organ. After releasing the forceps, renal reperfusion occurred and was visually confirmed by the return of the initial and natural color of the organ. Hemostasis was achieved, the kidney was repositioned, and the incision was closed in planes using 4-0 mononylon. In the immediate postoperative period, flunixin meglumine (2.5 mg/kg) was subcutaneously administered, and the animals were continuously monitored.

After 21 days of surgery, the RDAT group initiated its adaptation to an AT (swimming) protocol for 7 days; they gradually performed exercises without a working load until completing 30 min of swimming. The exercises were performed in 100-L tanks (75 × 85 × 40 cm^3^) at a temperature of 28°C-32°C.

After the 7-day adaptation period, the RDAT group was subjected to a high-intensity AT (HIAT) protocol. To determine the maximum training load, the animals were subjected to an incremental exercise test according to the protocol described by Cunha *et al.* ^6^ In this test, the rats were required to swim with an initial load corresponding to 1% of the body mass (BM) using pieces of lead of different sizes and weights to reach the weight appropriate for the exercise. The weights were attached to the distal part of the tail of the animals with hypoallergenic adhesive tape. The animals were removed from the water at every 3 min of swimming and rest for 1 min, and the exercise load was increased by 1% of the BM. This cycle was repeated until the animals reached exhaustion.

In the HIAT protocol, the training load was 85% of the maximum load achieved in the incremental test, and this value corresponded to 4.5% of the BM. The maximum load was adjusted weekly according to the BM of each animal. The weights and modes of attachment of the weights to the animals were the same as those used in the incremental test protocol. HIAT was performed five times a week for 6 weeks, and each training session lasted an average of 30 min. At the end of the protocol, the incremental test was performed again in the same manner as the initial test. The groups HS and RDS remained sedentary under daily care until the end of the training period of the RDAT group, when euthanasia was performed.

All animals were subjected to euthanasia by an intraperitoneal injection of ketamine and xylazine, both at 100 mg/kg. Laparotomy and exsanguination were performed by puncture of the hepatic portal vein. Blood samples were collected for biochemical analysis, and right gastrocnemius muscle samples were collected for morphometric analysis.

To measure serum creatinine, samples were centrifuged at 3,000 rpm for 10 min using an automatic refrigerated centrifuge (Sorvall, Suwanee, USA) for serum separation. The serum was kept under refrigeration (2°C-8°C) and later assayed using the UV enzymatic method.

The cross-sectional area (CSA) of gastrocnemius muscle fibers was used to establish a histological parameter of muscle adaptation to AT according to the method described by Brunnquell *et al.* ^7^ Muscle samples were collected and fixed in methanol and glacial acetic acid (3:1, v/v) for 70 min and embedded in paraffin. Each specimen was fixed on separate slide. Semi-serial sections (5-µm thickness, five sections per slide, and a 50-μm interval between sections) of each specimen were stained with hematoxylin and eosin. In CSA analysis, images of the tissue sections were not labeled to blind the examiner to group allocation. Five fields were captured per section on each slide, and 25 muscle fibers were measured per field, resulting in approximately 625 measurements per animal using an optical microscope (x100 magnification) coupled to a high-resolution camera. The images were analyzed using the Image Pro-Plus software version 4.0 (Media Cybernetics, Silver Spring, MD, USA), and the CSA was measured in square micrometers.

The results of weight, maximum load, and serum creatinine levels are presented as mean and standard error of the mean. Two-way ANOVA repeated measure with Tukey’s post-hoc test was used for the statistical analysis of weight (group factor, time factor and interaction). One-way ANOVA with Tukey’s post-hoc test was used for the statistical analysis of serum creatinine. Paired Student’s *t*-test was used for analyzing the maximum training load. Muscle morphometry data show the measurement of the CSA of the muscle fibers (in μm^2^) and were organized in absolute frequency of occurrence divided in class interval (with 500.00 μm^2^ each), totalizing 24 classes from 0 to 11500.00 μm^2^ and presented as histograms. P<0.05 were considered statistically significant.

## Results

The results of comparison of body weight, maximum load in incremental tests, and serum creatinine levels in the study groups are shown in Table 1.

**Table 1:** Body weight, maximum load, and serum creatinine levels in the study groups.

	HS	RDS	RDAT
Initial weight (g)	202.00 ± 4.44	204.00 ± 3.92	241.00 ± 5.88^a^
Pre-training weight (g)	321.00 ± 4.26	359.00 ± 5.45 ^b^	302.00 ± 6.10
Final weight (g)	418.00 ± 15.94	453.00 ± 6.19^c^	399.00 ± 6.97
Δ weight (g)	215.00 ± 15,05^d^	249.00 ± 19,49^d,e^	158.00± 4,61^d^
Maximum pre-exercise load (g)	_____	_____	19.72 ± 0.32
Maximum post-exercise load (g)	_____	_____	24.98 ± 1.16*
Serum creatinine (mg/dL)	0.55 ± 0.04	0.82 ± 0.04^#^	0.55 ± 0.04

HS: healthy sedentary group, RDS: renal disease sedentary group, RDAT: renal disease group. Δ weight = final weight- initial weight. All variables are shown as mean ± SE. Weight variables analysis - Two-Way ANOVA repeated measure with Tukey’s post-hoc test (p<0.0001 for time factor, p<0.004 for group factor and p<0.0001 for interaction) - Significant Tukey’s post hoc test: ^a^ Initial weight p<0,01 comparing HS x RDAT and p< 0,05 comparing RDS x RDAT; ^b^ Pre-training weight p<0,05 comparing HS x RDS and p<0,001comparing RDS x RDAT; ^c^ Final weight p< 0,05 comparing HS x RDS and p<0,001 comparing RDS x RDAT; ^d^ Δ weight p<0,001 comparing intra group; ^e^ Δ weight p<0,01 comparing between groups HS x RDS and RDS x RDAT. ^#^ One-way ANOVA with Tukey’s post-hoc test: p<0.05 comparing HS x RDS and RDS x RDAT. * Paired Student’s *t*-test: p < 0.05 comparing with maximum pre-exercise load.

The analysis of the incremental test results indicated a significant increase in the maximum load (p = 0.008) in the RDAT group at the end of the training period, with an increase of 26.67% in the final maximum load, and this result was compatible with the adopted training protocol.

Serum creatinine is a marker of renal function and was higher (p=0.0053) in the RDS group than in the other two groups. Nonetheless, there was no significant difference in serum creatinine between the HS and RDAT groups, which demonstrated that HIAT protected renal function.

The analysis of body weight shows that RDAT group presented significantly higher weight than the other groups at the beginning of the experiment, even though they were animals of the same age, from the same brood under the same daily care. All groups gained weight throughout the experiment as expected by the effect of growth and age. At the pre-training moment and at the end of the experiment the RDS group presented significantly higher body weight than the other groups. The RDAT group presented lower total weight and lower weight variation than RDS group at the end of the experiment but did not differ significantly from the HS group. This fact may be related to the reduction of renal function that results in fluid retention and can also be associated with the accumulation of body fat secondary to sedentarism. This effect was not observed in the RDAT group submitted to HIAT.

**Figure 1:**
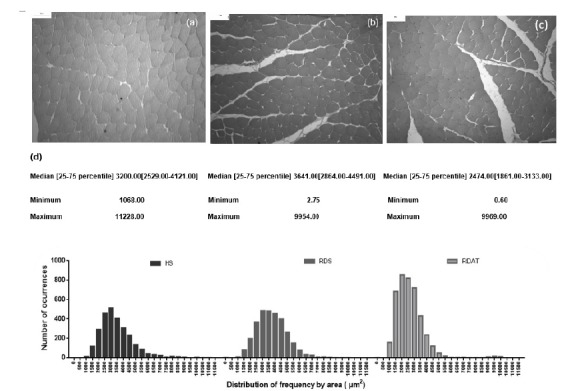
Histological and morphometric differences in the gastrocnemius muscle into study groups. **a**) healthy sedentary (HS); **b**) renal disease sedentary (RDS; **c**) renal disease aerobic training (RDAT); **d**) histogram of the frequency distribution of the cross-sectional area of the muscle fibers (µm^2^). Median values (25-75 percentile) and minimum and maximum values were added in the graph to indicate the changes (x100 magnification).

The frequency distribution of the CSA of gastrocnemius muscle fibers was significantly different between the groups (Fig. 1). The 50th percentile of CSA occurred in the seventh class (3000.00-3499.00 μm^2^) in the HS group, eighth class (3500.00-3999.00 μm^2^) in the RDS group, and fifth class (2000.00-2499.00 μm^2^) in the RDAT group. The cumulative number of muscle fibers in the 50th percentile in the groups HS, RDS, and RDAT was 515, 484, and 856, respectively. There was an increase in the frequency of observations of muscle fibers with smaller CSA in the RDAT group (4th, 5th, 6th and 7th class) and an absence of observation of muscle fibers with larger CSA (22nd and 23rd classes) in the RDS and RDAT groups. The CSA of largest muscle fibers observed in the RDS group was 9954.00 µm^2^ (belonging to 21th class), 9969.00 µm^2^ (belonging 21th class) in the RDAT group and 11228.00 µm^2^ (belonging to 23th class) in HS group.

## Discussion

Aerobic exercise has been shown to have several benefits, including improvement in physical capacity, cardiovascular function, and metabolic regulation^8^. In this study, the effect of HIAT on muscle tissue was assessed in an animal model of RD.

HIAT (swimming) improved exercise capacity in trained rats with RD by significantly increasing the maximum load supported during effort at the end of the training period. AT can reduce the effect of uremia on muscle tissue and increase muscle function, consequently improving exercise tolerance^9^.

The RDS group represented the progression of RD after 80 days of 10-min unilateral renal IR procedures. These animals exhibited significant changes in Cr levels relative to the other groups. Renal IR causes local and systemic changes as a consequence of oxidative stress, and it is well documented that acute kidney injury promotes changes in the kidneys that lead to acute renal failure and subsequent CKD to some degree due to oxidative stress during IR injury^10^. Oxidative stress plays an essential role in CKD progression because of the significant oxidation of proteins, carbohydrates, and lipids, leading to lipid peroxidation and the accumulation of glycation products that damage tissues, causing inflammation, endothelial lesion, glomerular lesion, and further renal ischemia, leading to severe damage to the renal environment^11^.

Another contributing factor in this context is the structural and functional changes that occur in kidneys subjected to unilateral IR procedures for short periods. Bazzano *et al.* ^5^ observed that 10-min unilateral renal IR in rats worsened urinary parameters (protein, urea, glucose, fractional excretion of potassium and urea, creatinine clearance, and activity of alkaline phosphatase and gamma-glutamyl transferase) 21 days after injury. Togoe *et al.* ^12^ found that 20-min unilateral renal IR in rabbits led to histopathological lesions of the renal tissue and increased serum creatinine 80 days after injury. The creatine results in our study are consistent with those reported by Bazzano *et al.* ^5^ and demonstrate that IR episodes, even for a few minutes, damage the kidneys and result in progressive RD. Slocum *et al.* ^13^ reported that after an abrupt decrease in the GFR (by minimum 30%), the increase in serum creatinine is delayed by days. The serum creatinine levels found in the RDS group demonstrate the progression of renal injury over time.

The lower serum creatinine detected in the RDAT group may be related to the effect of exercise on the sympathetic nervous system (SNS). The SNS is hyperactive in the early stages of CKD, causing changes in the tubular structure and stimulating the renin-angiotensin system, thereby leading to renal vasoconstriction, decreased blood flow, and reduction in GFR^14^. Aerobic exercise can restore SNS hyperactivity, suppress the renin-angiotensin system, increase blood volume, and consequently increase renal blood flow at rest and exercise, and ultimately reduce renal ischemia^15^.

Waldman *et al.* ^16^ observed that hypertensive rats subjected to running exercise exhibit sympathetic inhibition, lower excretion of aldosterone, and consequently lower blood pressure; however, renal function was not affected. Munõz *et al.* ^17^ found that aerobic exercise decreased the expression of angiotensin II receptors, suppressed the pro-inflammatory and pro-oxidant system, and reduced renal damage. Therefore, HIAT may help delay the progression of CKD by a direct effect on the sympathetic innervation of the kidneys and the consequent maintenance of the GFR, and this new hypothesis should be investigated because it may prevent the development of RD.

The body weight analysis suggest that having a renal disease and being sedentary contribute to weight gain due to fluid retention caused by a decrease in renal function added an increased of body fat secondary to sedentary lifestyle. This is reinforced by the results of lower final weight and lower weight variation found in the RDAT group when compared to the RDS group, without significant difference between RDAT and HS groups, and show the positive effects of physical exercise on renal function (discussed previously) and body composition, because HIAT increases energy expenditure which can justify the change in body composition. The literature shows that these effects are physical exercise intensity dependent and related to the release of catecholamines, growth hormone, oxygen consumption after exercise, lipoprotein lipase activity and improvement of the insulin response that usually affect the mobilization of triacylglycerol and in the oxidation rate, resulting in reduction of fat mass^18^. 

The morphometric analysis of the gastrocnemius muscle indicated that there was a decrease in the number of muscle fibers of larger CSA in rats with RD (RDS and RDAT) compared with that in the HS group. The RDAT group had a higher number of fibers of smaller CSA than the other groups. The morphometric changes in the RDS group indicated that muscle remodeling occurred in this group secondary to renal injury, which shows the occurrence of early muscle impairment even in cases of mild CKD. Considering that skeletal muscle is the main protein storage tissue in the body, the morphometric results suggest a significant reduction in contractile proteins due to an increase in muscle catabolism and/or decrease in muscle anabolism in RD^19^.

Muscular atrophy in CKD is primarily caused by the activation of protein catabolism. The uremic state, metabolic acidosis, inflammation, and oxidative stress activate mechanisms that stimulate protein catabolism. These changes lead to decreased proliferation and differentiation of satellite cells, insulin resistance, and activation of inhibitory factors of muscle tissue growth. These factors stimulate proteolysis via caspase 3 and the ubiquitin-proteasome system, leading to muscle atrophy^20^.

HIAT did not attenuate the loss of muscle fibers of larger CSA in the RDAT group. However, there was a higher number of muscle fibers of smaller CSA, of fourth to seventh class, indicating the adaptation of muscle fibers to HIAT. Sakas *et al* ^21^ have shown that the gastrocnemius muscle is composed of 55% type I (slow/oxidative) fibers and 45% type II (fast/glycolytic) fibers, and muscle atrophy in CKD affects both fiber types but predominantly IIa and IIx fibers, with a decrease in capillarization. The authors observed that AT promoted an increase in all fiber types in the gastrocnemius muscle.

The increased number of muscle fibers with smaller CSA may be related to the remodeling of type I fibers. Lepsen *et al.* ^22^ reported that AT stimulated the adaptation of muscle fibers to a more oxidative phenotype. However, the confirmation of this hypothesis requires specific morphological analysis of muscle tissue.

Knopka *et al*.^23^ argue that HIAT induces hypertrophy of muscle fibers I and IIa by regulating proteolytic mechanisms and increasing protein synthesis. The pathways involved in aerobic exercise-induced muscle hypertrophy include the upregulation of myostatin mRNA expression (satellite cell inhibitor), upregulation of the expression of PGC-1 coactivators, which control mitochondrial function, an increase in protein metabolism with concomitant reduction in catabolic factors, and an increase in muscle growth factors.

The levels of pro-inflammatory cytokines (IL6, TNF-α, and C-reactive protein) are increased in CKD, which stimulate the breakdown of muscle proteins. Moraes *et al.* ^9^ demonstrated that aerobic exercise attenuated muscle apoptosis via Akt signaling, reduced protein changes, thereby preventing proteotoxicity, and reduced cellular stress induced by chronic uremia.

Cardoso *et al.* ^24^ observed that high-intensity swimming exercise increased antioxidant levels in rats and prevented the increase in inflammatory markers. Miyagi *et al.* ^25^ found that aerobic exercise reduced the levels of tumor necrosis factor alpha (TNF-α) in rats with RD, thereby modulating the inflammatory response. These mechanisms may explain our results and will be analyzed in future studies.

Yoshida *et al.* ^26^ subjected rats with RD to AT for 5 weeks and found no significant differences in the CSA of muscle fibers or changes in protein synthesis and degradation. Organ *et al.* ^27^ demonstrated that aerobic exercise in rats with RD increases skeletal muscle catabolism via the upregulation of the atrogin-1 gene and increase in ubiquitination, without a concomitant increase in protein synthesis and the inadequate activation of satellite cells. Nonetheless, the effect of AT on RD remains controversial and needs to be further investigated.

Our results indicate that AT protected the kidneys, did not reverse the atrophy of muscle fibers of higher CSA, but increased the number of fibers of smaller CSA in the training group. These results are not conclusive but suggest that HIAT can be used to delay the loss of muscle mass in CKD because this complication occurs early and decreases the quality of life of the individual. However, biochemical analysis of specific markers should be performed to support the use of HIAT as a strategy to minimize the loss of muscle mass in RD.

## Conclusion

The results demonstrated that HIAT in an animal model of RD increased the number of muscle fibers of smaller CSA.
